# *Agrimonia eupatoria* Leaf Extract Attenuates Alcohol‐Induced Oxidative Stress, Ulcer and Alleviates Stomach Damage in Rats

**DOI:** 10.1002/fsn3.71203

**Published:** 2025-11-19

**Authors:** Suhayla Hamad Shareef, Nahla Kamal Asaad, Noor Ali Gheni, Derin Nabaz Fisal, Nabaz Fisal Shakir Agha, Ronak Tahr Ali, Mahmood Ameen Abdulla

**Affiliations:** ^1^ Department of Biology, College of Education Salahaddin University‐Erbil Erbil Kurdistan Region Iraq; ^2^ Department of Forensic Evidence, College of Science Al‐Kitab University Kirkuk Iraq; ^3^ Department of Animal Production, College of Agriculture University of Kirkuk Kirkuk Iraq; ^4^ Department of Biology, College of Education‐Shaqlawa Salahaddin University‐Erbil Erbil Kurdistan Region Iraq; ^5^ Department of Pharmacology, Erbil Medical Technical Institute Erbil Polytechnical University Erbil Kurdistan Region Iraq; ^6^ Department of Anesthesia, Erbil Medical Technical Institute Erbil Polytechnic University Erbil Kurdistan Region Iraq; ^7^ Department of Technical Radiology, College of Medical Technology Al‐Kitab University Kirkuk Kurdistan Region Iraq; ^8^ Medical Analysis Department, Applied Science Faculty Tishk International University Erbil Kurdistan Region Iraq

**Keywords:** *A. eupatoria*, *endogenous enzymes*, *gastric ulcer*, *histology*

## Abstract

*Agrimonia eupatoria* commonly utilized in traditional medicine in treating various ailments has not been investigated for its impact on gastric ulceration in rats. This study aims to examine the gastroprotective effects of *A. eupatoria* leaf extract on acute gastric mucosal damage induced by absolute ethanol in *Sprague Dawley* rats. Thirty rats were randomly assigned to five groups: negative control, ulcer control, omeprazole, and two experimental groups. Macroscopic examination revealed severe stomach mucosal injuries and reduced stomach mucus secretion and stomach pH in the ulcer control rats. Rats pre‐treated with *A. eupatoria* extract exhibited a significant reduction in ulcer area, increased mucus production, and an increase in stomach pH compared to the ulcer control group. Histology of rats pre‐nourished with *A. eupatoria* extract using hematoxylin and eosin (H & E) stains importantly decreased gastric lesions, ulcer areas, edema, and white blood cell permeation of the submucosal stratum. Likewise, treated groups showed increased intensity of periodic acid‐Schiff (PAS) staining in the stomach epithelium compared to the ulcer control group, indicating enhanced mucosal protection. In stomach homogenate, *A. eupatoria* established a significant increase in superoxide dismutase (SOD), and catalase (CAT) activities as well as significantly reduced malondialdehyde (MDA) levels. In conclusion, *A. eupatoria* extract documented gastroprotective influence, reduction in ulcer area, rise in pH, and mucus excretion, increased SOD and CAT, and reduced MDA level.

## Graphical abstract

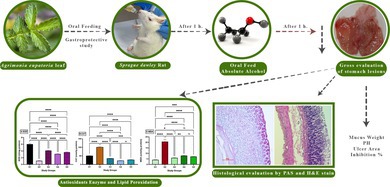

## 1 Introduction

Stomach ulcers represent a prevalent digestive system disease in the human population, with an occurrence rate of 2.4% and 6.1% in Western and Chinese populations, respectively (Fu et al. 2021). A peptic ulcer, a lesion within the digestive system induced by gastric acid, commonly grows in the stomach or the proximal duodenum (Kuna et al. 2019; Shareef, AL‐Medhtiy, et al. 2022). Peptic ulcer disease (PUD) is widespread, with a cumulative incidence that ranges from 5% to 10% in ordinary people and a yearly occurrence rate from 0.1% to 0.3% (Lanas and Chan 2017). In 2019, the prevalence of this disease reached nearly 8 million documented cases (Xie et al. 2022).

Among the agents triggering tissue necrosis, alcohol stands out as a prevalent factor in gastric damage (Shin et al. 2021). Ethanol detrimentally affects the mucosa of the gastric by disrupting the equilibrium of the stomach's mucosa barrier, enabling the permeation of stimulating cells and the termination of inflammatory factors (Li et al. 2023; Ibrahim et al. 2022). Damage to gastric mucosal epithelial cells shows a vigorous part in expanding gastric ulcers, a common characteristic of gastrointestinal ulceration (GU). Clinical strategies involving the use of proton pump inhibitors, such as omeprazole, have been employed to reduce GU. However, the prolonged use of chemical medications, such as omeprazole is correlated with adverse reactions and an elevated recurrence rate (Shareef, AL‐Medhtiy, et al. 2022; Liu et al. 2022). Accordingly, a harmless and efficacious organic compound is required to avoid and address ethanol‐induced gastric injury. Presently, researchers are exploring the application of natural substances, including terpenoids, flavonoids, and polysaccharides extracted from plant sources, as medicinal or dietary boosters (Zhang et al. 2021; Shareef, Hadi, and Abdulla et al. 2022; Shareef, Saeed, and Majeed et al. 2023; Shareef et al. 2025).

Proton pump inhibitors (PPIs) and histamine receptor type‐2 antagonists, commonly utilized in medical practice, have been associated with adverse effects and numerous drug interactions (Golbabapour et al. 2018; Sherif 2019). Consequently, the imperative to address these issues has spurred research endeavors, leading to investigations into novel bioactive compounds derived from natural sources (Blunt et al. 2018; Taha et al. 2023).

Genus *Agrimonia* L. plants, as well as the commonly known *Agrimonia eupatoria*, serve as rich sources of ellagitannins and flavonoids. *A. eupatoria* is predominantly utilized for therapeutic applications, commonly administered in brew methods (Kuczmannová et al. 2015; Karlińska et al. 2021). Concerning agrimony, the herbal ingredient, including the blossom‐harvested plant parts, spans from the beginning of July to the middle of August. The maturation of agrimony fruits (achenes) shortly follows the flowering stage (Rutkowski 1998). This practical consideration implies that during the highpoint phase, bunches comprising both blossoms and maturing fruits coexist with leaves and stalks. *A. eupatoria* demonstrates resistance to syndromes, and periodic water scarcity, and does not necessitate the use of agrochemicals (Karlińska et al. 2021).

Specifically, considerable in vitro and in vivo research has been dedicated to discovering two distinct collections of polyphenols—namely, ellagitannins and flavonoids—that are prevalent in the genus *Agrimonia* L. (Alirezalu et al. 2018). This prompts a compelling investigation of novel practical applications for incorporating *Agrimonia* L. plants into the food industry due to their potential as abundant sources of phytocomponents, as well as polyphenols.

Scientific studies have highlighted the considerable pharmaceutical and biological potential inherent in plant‐based products derived from the aerial components of *A. eupatoria* L. Recent findings, such as the documented in vitro anticoagulant activity (Tsirigotis‐Maniecka et al. 2019), feature this potential to the conjugates of pectin‐like polysaccharides and macromolecular polyphenolic matrices. Various small molecule polyphenols, as well as flavonoids, phenolic acids, coumarins, tannins, and terpenoids, derived from *A. eupatoria* , have displayed health‐promoting effects primarily owing to their recognized capacity for scavenging free radicals (Paluch et al. 2020; Malheiros et al. 2022). Previous studies highlight the abundance of flavonoid glycosides in *A. eupatoria* tissues, namely luteolin, acacetin, apigenin, quercetin, kaempferol, kaempferide, including rutin (Karlińska et al. 2021), along with flavonols such as catechin, epicatechin and its polymers, myricetin, isorhamnetin, and phenolic acids, as well as p‐coumaric, vanillic, gentilic, p‐hydroxybenzoic, and hydroxycinnamic acids (Tsirigotis‐Maniecka et al. 2023). In addition to these promising outcomes, a thorough investigation of the impact of *A. eupatoria* leaf extract on acute gastroprotective properties through trial studies is further required. Therefore, this study sought to examine the gastroprotective efficacy of the ethanolic extract derived from *A. eupatoria* against stomach ulcers induced by ethanol in rodent models.

## 2 Materials and Methods

### 2.1 Plant Collection

**FIGURE 1:**
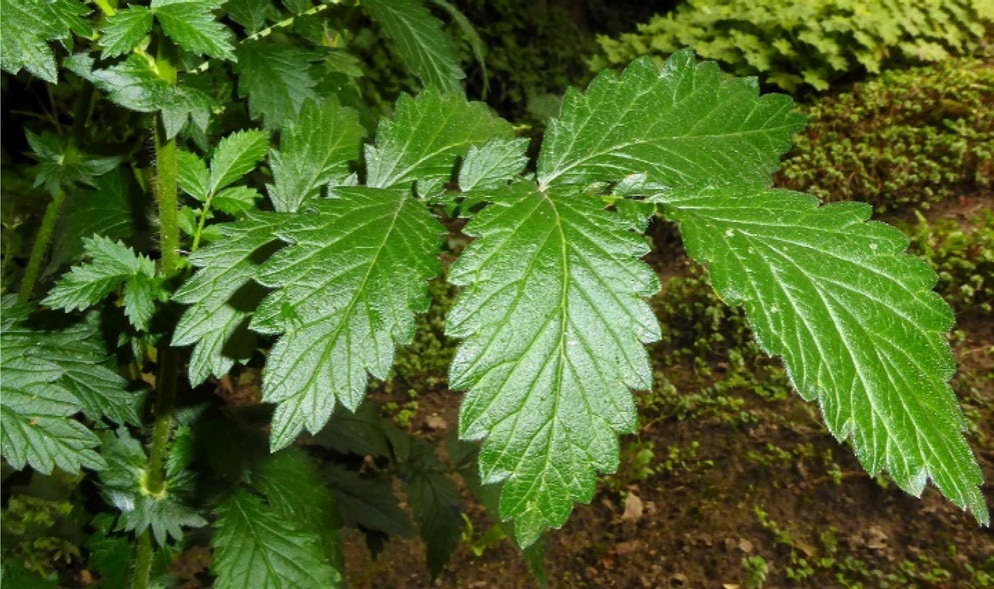
Morphology of *Agrimonia eupatoria* leaf.

*A. eupatoria* was discovered in Zine‐Asterokan Mountain in the Erbil region (Figure 1). This plant's authentication was carried out by the botanist Dr. *Abdul‐Hussain Al‐Khayat*, taking into account shared characteristics discovered in Iraq. Voucher No. 6735 was issued by the Herbarium of the Biology Division at the Science College, Salahaddin University.

### 2.2 Plant Extraction

The leaves of *A. eupatoria* were subjected to a drying process in the absence of direct sunlight, and subsequently, 100 g of the dried material was immersed in 95% ethanol (900 mL) for 5 days. Extraction of the solution was made by utilizing the Rotary Evaporator, obtained from Chemo Pharm (Mughrabi et al. 2011). For assessing anti‐ulcerative action contrary to ethanol—convinced stomach's epithelial damage, the powder derived from *A. eupatoria* leaves liquefied in 10% Tween 20, with dosages of 250 and 500 mg/kg established ingestion, adhering to the procedures outlined in the previous study (Abdullah et al. 2012).

### 2.3 Ethical Permission

The rats, weighing from 215 to 230 g, were purchased from Experimental House at Cihan University‐Erbil, with ethical approval under the reference number Biology/15/10/2022/MAA. The experiments were conducted following the established ethical standards for laboratory animal care and use, according to the guidelines provided by the “Principles of Laboratory Animal Care.”

### 2.4 Gastric Ulcer

#### 2.4.1 Omeprazole

In this study, omeprazole which is a standard antiulcer medication available at a pharmacy, was utilized. Omeprazole was solubilized in a 10% Tween 20 solution and then administered by mouth at a dosage of 20 mg/kg (Mughrabi et al. 2010).

#### 2.4.2 Ethanol‐Induced Gastric Ulceration

A total of five groups, consisting of 6 rats per group, were formed. Before the experimentation, a 24‐h fasting period (food but not water) was implemented. The animals were kept in cages with wire bottoms.

Experimental design and treatment groups:

**Table:** 

Animal groups	Treatment	Administration (orally)	Follow‐up treatment (orally)
Group 1 (Normal)	10% Tween 20	5 mL/kg	10% Tween 20 (5 mL/kg)
Group 2 (Ulcer)	10% Tween 20	5 mL/kg	Absolute alcohol (5 mL/kg)
Group 3 (Omeprazole)	Omeprazole	20 mg/kg (5 ml/kg)	Absolute alcohol (5 mL/kg)
Group 4 ( *A. eupatoria* 250 mg/kg)	*A. eupatoria* extract	250 mg/kg (5 mL/kg)	Absolute alcohol (5 mL/kg)
Group 5 ( *A. eupatoria* 500 mg/kg)	*A. eupatoria* extract	500 mg/kg (5 mL/kg)	Absolute alcohol (5 mL/kg)

After 60 min, group 1 received 10% Tween 20; groups 2–5 received absolute alcohol (5 mL/kg) orally (Ibrahim et al. 2022). An hour later, all animals were anesthetized with Xylazine and Ketamine, followed by cervical disarticulation and surgical removal of the stomach (Wasman et al. 2010; Shareef 2024).

#### 2.4.3 Gross Evaluation of Gastric Mucosa

Following excision along the greater curvature, stomachs were rinsed with ice‐cold buffered saline. The epithelial surface was examined under a dissecting microscope (1.8× magnification) for hemorrhagic lesions appearing as elongated red bands paralleling the stomach's longitudinal axis. Ulcer areas were quantified using a grid comprising 2 mm × 2 mm squares, counting squares overlying lesions to determine the extent of mucosal damage (Ibrahim et al. 2022; Shareef, Hadi, and Abdulla et al. 2022).

Ulcer area calculation and inhibition percentage:

– Ulcer Area (UA, mm^2^): Summation of affected squares × 4 × 1.8.
– UA (mm^2^) = Number of small squares × 4 × 1.8.
– Inhibition Percentage (I%): = (UA control– UA treated/UA control) × 100 (Shareef, Hadi, and Abdulla et al. 2022).

#### 2.4.4 Assessment of Stomach's Fluid Sourness and Mucus‐Contented

Greater curvature of every stomach cut, then gastric contents underwent analysis through pH meter titration employing 0.1 N NaOH. An assessment of both acids gratified by stomach epithelia was performed to quantify the acidity of the gastric juice (Anuar 2009).

### 2.5 Histological Evaluation of Gastric Lesions

#### 2.5.1 Hematoxylin and Eosin Staining

The specimens from the gastric wall underwent fixation in 10% buffered formalin, followed by processing and paraffin embedding. Subsequently, the stomach sections were crafted with a width of 5 μm then subjected to H & E staining to aid in the assessment of histological features and tissue architecture (Mahmood et al. 2005).

#### 2.5.2 Mucosal Glycoprotein Staining

Evaluation of mucosal glycoprotein formation, stained with PAS, was performed according to the instructions from the manufacturer, using the Sigma periodic acid–Schiff commercial kit (Shareef, AL‐Medhtiy, et al. 2022). PAS staining was employed to evaluate differences in glycoproteins, including both acidic and basic types. The mucus produced was photographed and observed using a compound microscope (Hussaini et al. 2013).

### 2.6 Antioxidant Activity of Gastric Homogenate

#### 2.6.1 Preparation of Homogenate

The samples of gastric tissue underwent thorough washing with ice‐cold PBS. Subsequently, the homogenate (10% w/v) was prepared using a 50 mM PBS solution with a homogenizer (Omar et al. 2017). Homogenates were subjected to centrifugation lasting 15 min at 10,000 rpm, employing a refrigerated centrifuge. The resulting supernatant was utilized to evaluate the antioxidant activities and MDA levels (Shareef, AL‐Medhtiy, et al. 2022).

#### 2.6.2 Measurement Antioxidant Actions Gastric Homogenates

Activities of SOD and CAT in the homogenized gastric tissues were evaluated through marketable kits. Techniques recommended by the manufacturer were adhered to in assessing the gastric tissue supernatant activities per sample (Mahmood et al. 2005; Alqahtani et al. 2024).

#### 2.6.3 Quantities Lipid Peroxidation (MDA) Gastric Homogenate

The assessment of lipid peroxidation in a mucous sheath of stomach tissue homogenates is carried out utilizing a profit‐making kit (Mahmood et al. 2004).

### 2.7 Statistical Analysis

Each value in this study was stated as means ± standard errors of the mean. Arithmetical analysis to measure important differences among groups was made utilizing the Graph Pad Prism 9, and SPSS statistical software program version 20, employing a one‐way analysis of variance monitored via the post hoc Tukey's multiple appraisal assessment. The consequence amount of *p <* 0.05 is regarded as an indicator of substantial.

## 3 Results

### 3.1 Antiulcer Study

#### 3.1.1 Gross Evaluation

**FIGURE 2:**
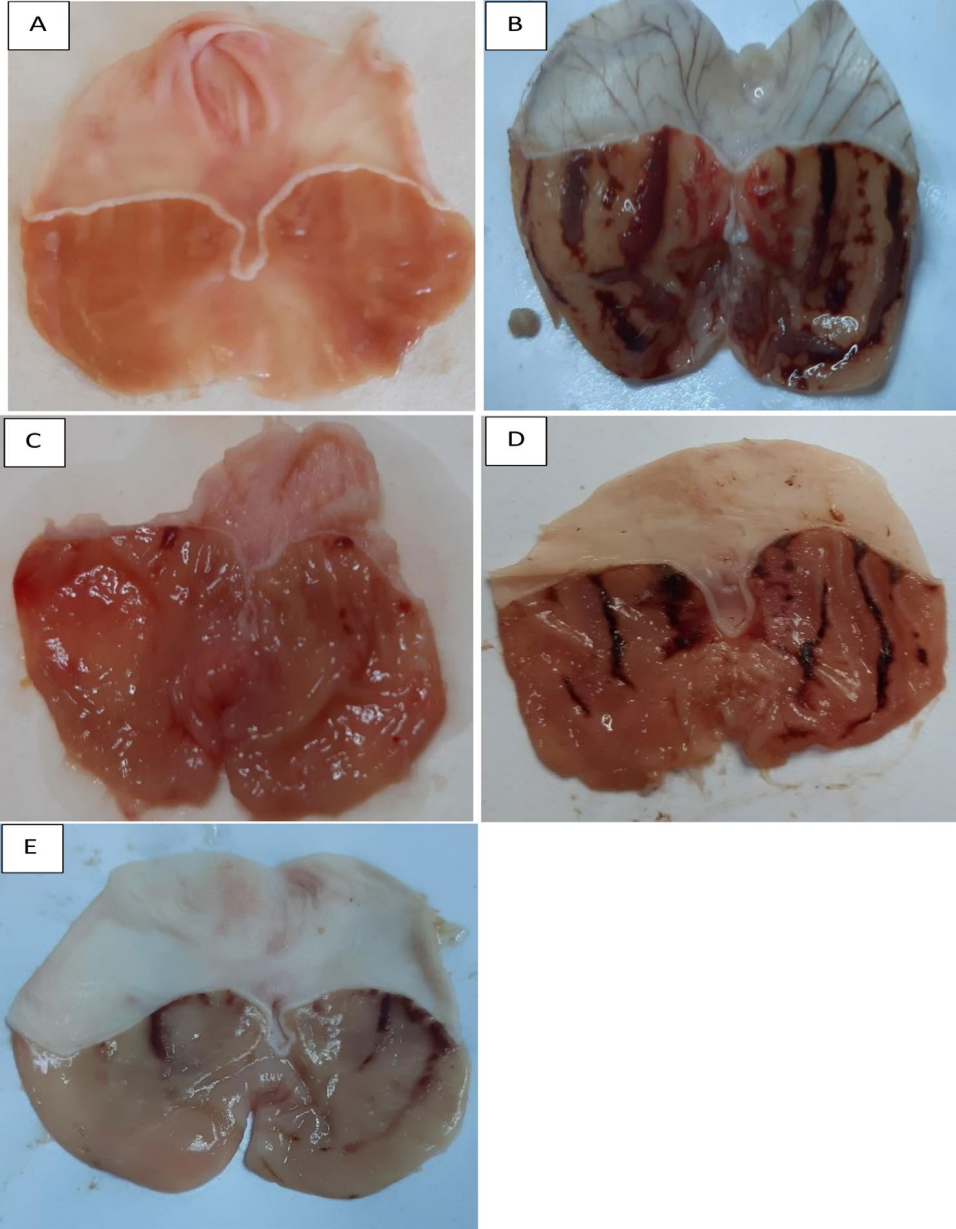
The effect of *A. eupatoria* gross presence gastric epithelia with ethanol‐induced gastric epithelial lesions was assessed. The negative control (A) displayed no macroscopic damage to the stomach epithelium; however, the ulcer group (B) demonstrated basic wounds. Omeprazole (C) presented slight disturbances in the superficial mucosa of the stomach epithelium. Experimental groups (D, E), administered 250 mg/kg and 500 mg/kg, respectively, indicated disruptions of the epithelium surface ranging from moderate to mild in the gastric mucosa.

The findings of this study showed a significant reduction in UA in Sprague Dawley rats pretreated with the ethanol extract of *A. eupatoria* leaf, contrasted with the ulcerated group (Figure 2, Table 1). The inhibition percentage of UA exhibited a dose‐dependent increase in rats pre‐administered with *A. eupatoria* extract.

**TABLE 1:** Assessment of *A. eupatoria* extracts' effects on gastric mucus, pH, ulcer area, and inhibition percentage of ulcer area.

Animals groups	Pre‐treated 5 mL/kg	Secretion weight (g)	pH acidity	UA (mm^2^)	I%
G1 Usual control	10% Tween 20	2.25 ± 0.15^a^	7.03 ± 0.33^a^	—	—
G2 Ulcer control	10% Tween 20	0.72 ± 0.25^b^	2.73 ± 0.25^b^	711 ± 27.17^b^	—
G3 Omeprazole	20 mg/kg	1.83 ± 0.04^a^	5.70 ± 0.55^a^	88 ± 15.25^a^	87.62
G4 *A. eupatoria*	250 mg/kg	1.54 ± 0.16^a^	5.64 ± 0.42^a^	155 ± 18.45^a^	78.16
G5 *A. eupatoria*	500 mg/kg	1.74 ± 0.16^a^	5.68 ± 0.45^a^	123 ± 11.27^a^	82.63

*Note:* Mean value ± SEM (*n* = 6). a,b Values indicated by different superscripts within the same column are significantly different according to Tukey's honestly significant difference test at a *p* < 0.05 significance level.

#### 3.1.2 Gastric Mucus Content and Acidity

The ulcer rat group in Table 1 displayed the least content of gastric mucosa mucus. On the other hand, animal groups that received pre‐treatment with G4 (250 mg/kg) and G5 (500 mg/kg) *A. eupatoria* extract depicted statistically momentous upsurge in secretion heaviness (g) compared to the ulcer group (G2). In addition, the pre‐treatment with *A. eupatoria* extract (G4 and G5) caused a substantial elevation in gastric contents' pH than the ulcer group (G2).

### 3.2 Measure Stomach Antioxidants Enzyme and Lipid Peroxidation (MDA)

**FIGURE 3:**
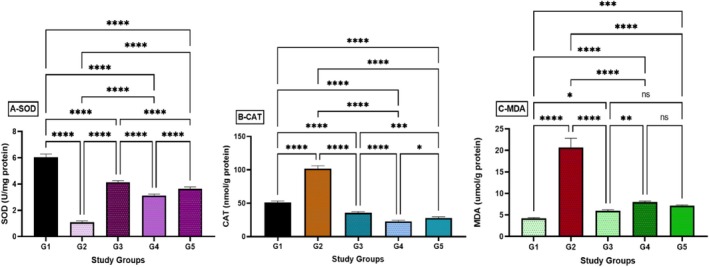
Effects of *A. eupatoria* leaf on antioxidant enzyme actions (SOD and CAT) and MDA quantity or level in the stomach. Information stated as mean ± SEM. Mean between groups (*n* = 6 rates/group) demonstrates substantial modification.

Ulcer rats displayed a considerable decline in endogenous enzyme activities related to antioxidants, explicitly SOD and CAT. As shown in Figure 3, rats that were pre‐treated with *A. eupatoria* extract exhibited an escalation in altogether antioxidant accomplishments compared to ulcer rats. Moreover, significantly higher SOD enzyme activities measuring 250 and 500 mg/kg *A. eupatoria* extract compared to the ulceration animals were depicted in Figure 3. A significant increase in CAT activity was seen in rats administered *A. eupatoria* leaf ethanol extract compared with the ulcer control rats. Figure 3 data for SOD and CAT enzyme activities in gastric mucosal homogenates displayed a marked upsurge in animals pre‐treated with *A. eupatoria* leaf ethanol extract and omeprazole, relative to G2. Conversely, Figure 3 showed significantly lower MDA levels in the *A. eupatoria* leaf ethanol extract in groups (G4–G5) than those in the ulcer control group (G2).

### 3.3 Histological Evaluation of Gastric Lesions

#### 3.3.1 Hematoxylin and Eosin Stains and PAS Stain

**FIGURE 4:**
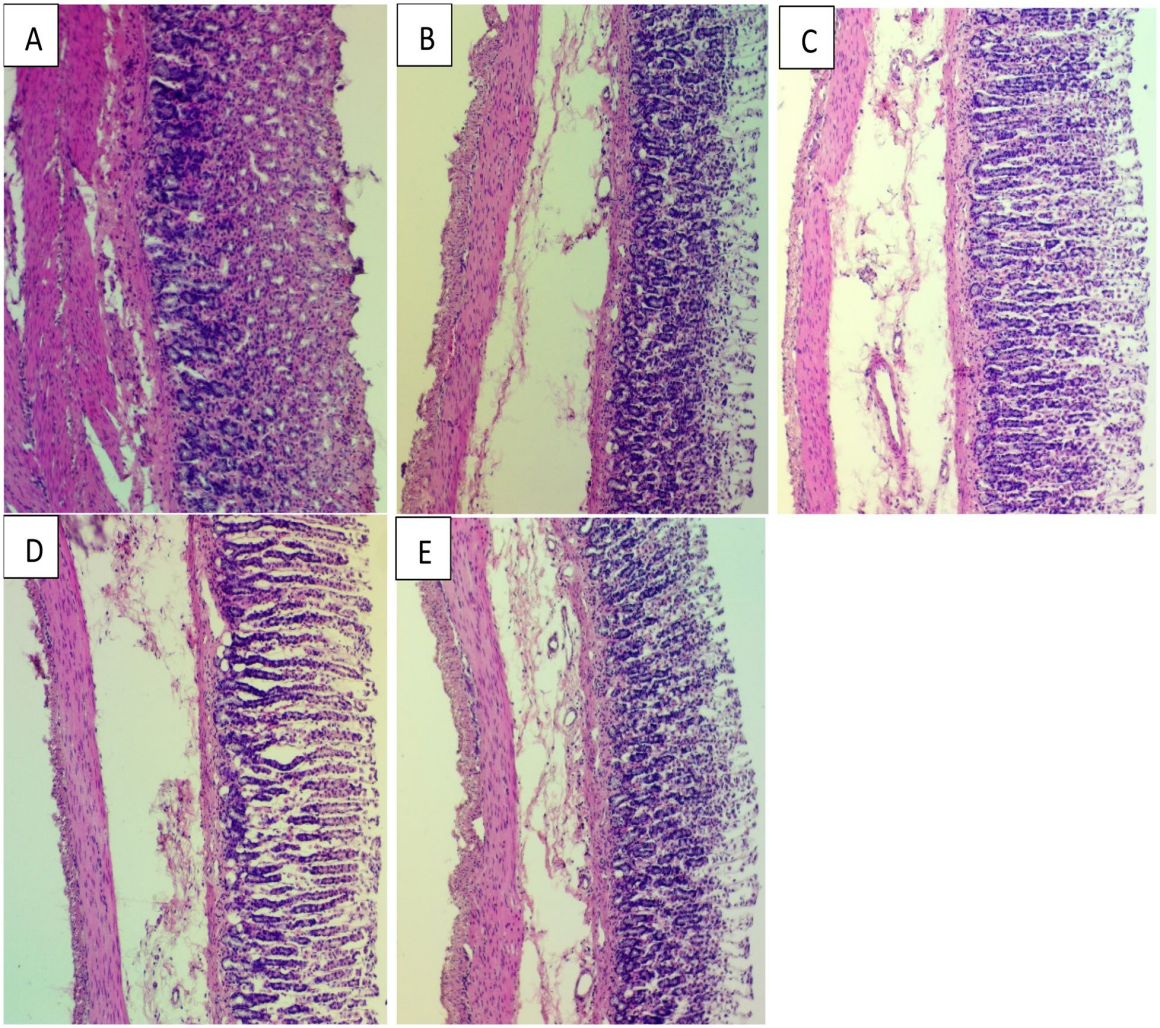
Assessment of the effect of *A. eupatoria* on the histological presence of gastric mucosa in alcohol‐induced stomach ulceration rats. (A) The normal group demonstrated the typical histological structure of stomach mucosa. (B) The ulcer control group demonstrated major structural damage to stomach epithelia, accompanied by edema and leukocyte permeation in the submucosal layer. (C) The omeprazole group (20 mg/kg) displayed minor injury to the gastric mucosa. (D) *A. eupatoria* 250 mg/kg showed moderate damage to the gastric mucosa. (E) *A. eupatoria* 500 mg/kg represented relatively mild injury to the gastric mucosa (H&E stain, 10×).

**FIGURE 5:**
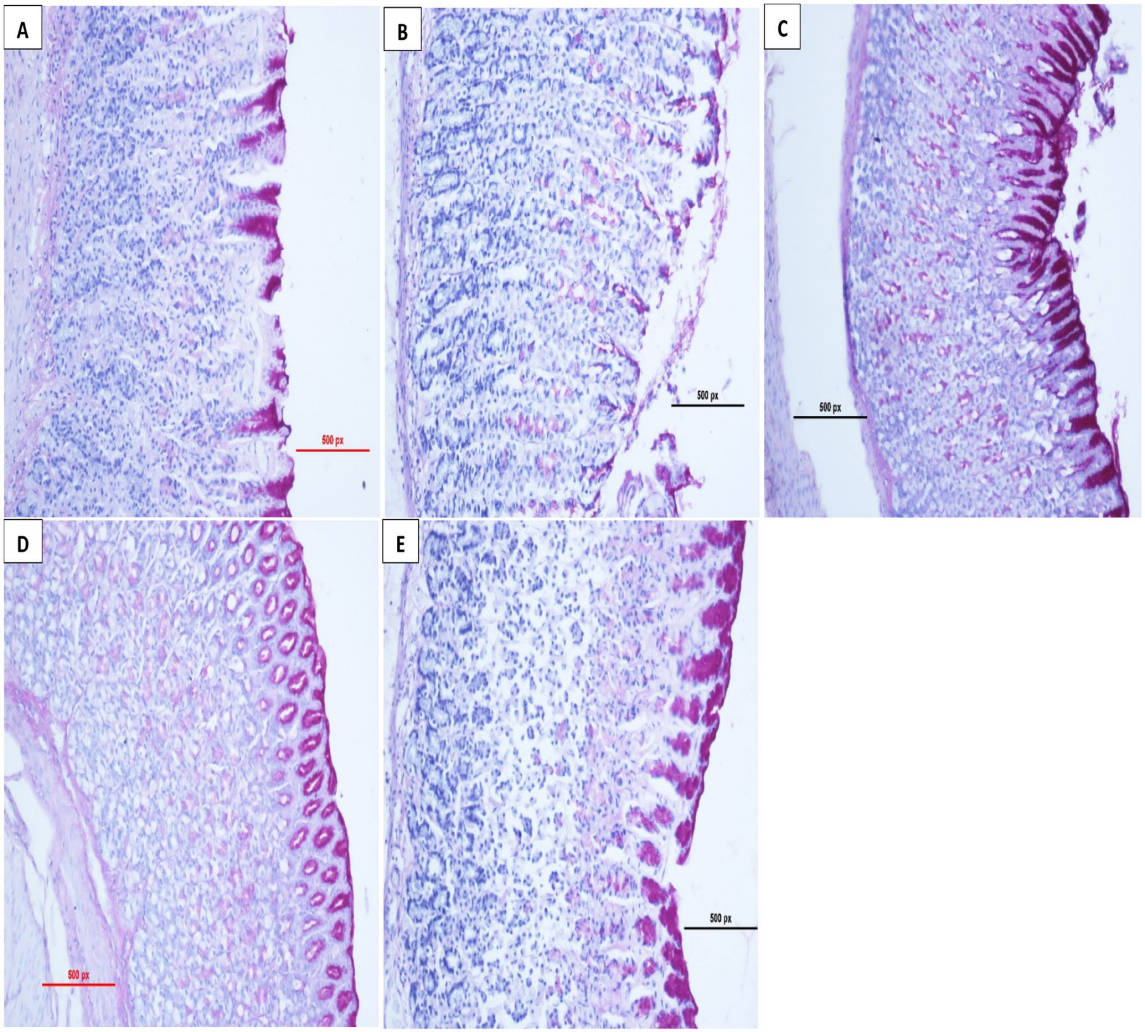
Assessment of the effect of *A. eupatoria* on the microscopic examination of PAS glycoprotein stains gastric mucosa alcohol—induce stomach ulceration rats. (A) The normal group showed a typical gastric mucosal structure and the absence of injury. (B) The ulcer control group displayed major injury to the gastric mucosa, along with a low PAS stain (magenta color). (C) In the omeprazole group (20 mg/kg), there was mild injury and an augmented concentration of PAS stains in stomach epithelia. (D) *A. eupatoria* 250 mg/kg indicated low to moderate injury of the gastric mucosa as well as a mild PAS stain. (E) *A. eupatoria* 500 mg/kg presented mild damage to the stomach mucosa with a moderate PAS stain (PAS stain, 20×).

Histology examination revealed extensive gastric mucosal damage in the control group of rats subjected to ulceration. In addition, this particular group of animals displayed necrotic graze stomach epithelia, indicating substantial WBC penetration besides submucosal layer edema, as shown in Figure 4. In contrast, animals that received pre‐treatment with *A. eupatoria* leaf ethanol extract demonstrated a relatively heightened defense stomach epithelium along with absent infiltration of inflammatory cells and edema (Figure 4). *A. eupatoria* extract show cased defensive properties following dosage levels, indicating markedly improved preservation of stomach epithelia. Pre‐fed investigational groups, gastric mucosa displayed steady elevation in PAS stain strength via an accumulation of magenta color epithelial cells stratum contrasted with the ulcerated collection, relying on the dose (Figure 5). Conversely, magenta staining was depicted as reduced and less abundant in gastric epithelium ulcer group induced with ethanol.

## 4 Discussion

*A. eupatoria* is a macromolecular complex consisting of polysaccharide and aglycone components, with the latter exhibiting a polyphenolic structure rich in dimethoxyphenyl subunits (Tsirigotis‐Maniecka et al. 2019; Pawlaczyk et al. 2011). Characterization revealed predominantly phenolic and carbohydrate constituents with a minor protein component (~1%) (Tsirigotis‐Maniecka et al. 2019). Polyphenols are valued for their antioxidant properties and free radical scavenging, protecting against oxidative stress via mitigation of reactive oxygen species (ROS) (Pignatelli et al. 2000). Given *A. eupatoria* 's structural diversity, encompassing hydrophobic and hydrophilic elements, its antioxidant capacity was assessed using assays suitable for both types of compounds, highlighting its potential to scavenge free radicals (Tsirigotis‐Maniecka et al. 2023).

*A. eupatoria* extracts demonstrate significant antioxidant activity, effectively neutralizing 2,2‐diphenyl‐1‐picrylhydrazyl (DPPH), hydroxyl, and superoxide anion radicals, with potency influenced by extraction solvent polarity (Gurita et al. 2018; Venskutonis et al. 2007). A methanol extract showed the highest antioxidant capacity, correlating with bioflavonoid content (Kubínová et al. 2012). *A. eupatoria's* antioxidant efficacy was quantified via total equivalent antioxidant capacity (TEAC) values, exhibiting the ability to scavenge ABTS and DPPH radicals (TEAC = 0.65; IVA = 200 μg/mL for ABTS) and reduce Cu^2+^ to Cu^+^ (Tsirigotis‐Maniecka et al. 2023). The antioxidant mechanisms likely involve hydrogen atom transfer (HAT) and single‐electron transfer (SET), suggesting *A. eupatoria* mitigates oxidative stress effectively. The polysaccharide fraction, including arabinogalactan and rhamnogalacturonan, contributes to the overall reducing properties, highlighting the interplay of phytochemical constituents in *A. eupatoria's* antioxidant profile (Tsirigotis‐Maniecka et al. 2019, 2023).

The findings of this study indicate that *A. eupatoria* extract possesses robust antioxidant actions besides free radical scavenging influence. Consistent with these results, herbal medicine displays antioxidant efficacy and leads to antioxidation, as discovered by previous studies (Golbabapour et al. 2018; Shareef, Al‐Medhtiy, et al. 2023).

This study has revealed that ethanol triggers profound disruption to the mucosa of the stomach, causing a decrease in bicarbonates and mucus release, and an elevation in gastric content acidity. These findings are consistent with previous studies (Shareef, Haldi, and Abdulla et al. 2022; Al‐Wajeeh et al. 2017). Chemicals, particularly alcohol, can compromise the defense mechanisms of the stomach by generating lesions in the stomach epithelium and varying the vascular layers' penetrability, causing edema. The initiation of gastric lesions by alcohol (ethanol) is able to take place through numerous mechanisms, including the lessening of gastric motility, a reduction in gastric pH, mucus, and bicarbonate secretion (Ibrahim et al. 2022; Taha et al. 2023).

The results of this study further show that *A. eupatoria* extract significantly reduces the secretion of gastric acid. This finding is consistent with previous studies (Shareef, Haldi, and Abdulla et al. 2022; Alhejaily et al. 2023). The secretion of gastric acid contributes to gastric ulcers. An increase in gastric mucus identified in this study implies it may lead to gastro‐protective effects of *A. eupatoria* extract. A layer of stomach secretion is acknowledged for its essential function in preserving the mucosa from internal threats, namely acid and pepsin, serving as a mediator in mucosal restoration (Taha et al. 2023). The findings of the current study indicate that severe damage to the gastric mucosa results from the oral administration of ethanol, bringing forth vascular endothelium disturbance, enhanced vascular penetrability, edema, and leukocyte permeation of the submucosal layer. Animals administered *A. eupatoria* extract displayed significantly preserved gastric epithelium, and this finding is in line with previous studies (Alhejaily et al. 2023; Salga et al. 2017).

Oxidative pressure denotes disproportion among generations besides the elimination of ROS within matters. This imbalance, due to stress conditions such as ethanol ingestion, can exacerbate tissue injury and damage to the gastric mucosa. The heightened production of harmful free radicals by gastric tissues under stress conditions including an inadequate or imbalanced presence of endogenous antioxidants in the gastric environment, can result in the occurrence and pathogenesis of gastric ulcers (Ibrahim et al. 2022; Moawad et al. 2019).

The findings from the current study reveal that the application of ethanol leads to the disturbance of stomach mucosa coating, which preserves the stomach's mucosal itself, in addition to lessening protective factor action, namely SOD and CAT enzymes. Furthermore, an escalation in microvascular penetrability lipid peroxidation, and cell rind within gastric mucosa is revealed in this study. Consequently, it may be suggested that *A. eupatoria* extract meditates a protective impact on the gastric mucosa through a scavenging mechanism (Gwaram et al. 2016; Gilani et al. 2022). Moreover, *A. eupatoria* extract showed its ability to protect the cell membrane by elevating the SOD and CAT activities, from ROS attack. ROS appears to be one of the primary adverse actions caused by ethanol in gastric cells (Salaheldin et al. 2022). On the contrary, a substantial lessening in the MDA levels was depicted in rats that received pre‐treatments with *A. eupatoria* extract, which might be caused by a decrease in oxidative gastric injury (Alhejaily et al. 2023; Alomair et al. 2022). These discoveries are in line with previous studies (Shareef, Hadi, and Abdulla et al. 2022; da Luz et al. 2021).

MDA is regarded as a well‐established subordinate polyunsaturated fatty acid molecule and acts as the primary signal in evaluating lipid peroxidation within tissues (Ibrahim et al. 2022; Golbabapour et al. 2018; Ahmed et al. 2024). In this study, the oral management of ethanol results in substantial oxidative stress in ulcerative rats (positive controls), as signified by a decrease in endogenous antioxidants (SOD and CAT) in addition to an increase in MDA contents within stomach tissue homogenate.

The inadequacy of the submucosal region of the stomach mucosal coat besides the decrease in WBCs permeation through the stomach barrier slices was seen in rats pre‐treated with *A. eupatoria* in this study. Edema plus hemorrhage grazes the epithelial stratum within an ulcerated group are regarded as signs of ethanol impairment. Results are consistent with the findings of past investigations (Li et al. 2023; Taha et al. 2023). The assessment of PAS stain in the current trial indicated that *A. eupatoria* excerpt increased glycoprotein in the gratified stomach epithelia, manifested by a magenta color. Increased mucus formation serves as a sign of local gastric mucosal defense, in line with the findings of the previous studies (Shareef, Hadi, and Abdulla et al. 2022; Hassan et al. 2023).

In the assessment of PAS staining, a marker of mucopolysaccharide secretion and a vital element of gastric mucus, the present data discover that *A. eupatoria* supplementation increases the glycoprotein content. This increased glycoprotein content functions as a defensive barrier for gastric tissues, protecting numerous epithelial damaging factors, namely toxins, microbes, and gastric acids. According to the PAS technique protocols, higher PAS stains detected in stomach tissues imply augmented gastric mucus secretions. The ulcer control rats displayed significantly lower PAS stain presence in their gastric tissues; however, animals treated with omeprazole or *A. eupatoria* presented increased PAS stain expression, indicative of glycoprotein content that facilitates the formation of a protective barrier, thereby minimizing gastric damage (Alhejaily et al. 2023; Shareef et al. 2025). The anti‐ulcerogenic activity of *A. eupatoria* extract comprises the scavenging of free radicals, suppression of inflammation, control of apoptosis, and maintenance of fibroblast growth factors, conjointly advancing the healing of gastric ulcers.

*A. eupatoria* demonstrates anti‐inflammatory properties (Huzio et al. 2022), modulating immune responses by suppressing pro‐inflammatory cytokines and enhancing anti‐inflammatory cytokines. The plant influences nitric oxide (NO) regulation and augments the expression and activity of key antioxidant enzymes including superoxide dismutase, catalase, and glutathione, complementing its direct free radical scavenging capacity. *A. eupatoria* mitigates liver damage through inhibition of TLR‐4 signaling pathways. Intestinal effects include inhibition of α‐glucosidase, leading to reduced glucose absorption. Additionally, extracts of *A. eupatoria* exhibit selective cytostatic activity against tumor cells, sparing normal cells, highlighting its potential therapeutic applications (Paluch et al. 2020).

Pro‐inflammatory cytokines, products of inflammatory processes, are regulated by transcription factors like NF‐κB, which plays a pivotal role in controlling the expression of cytokines including IL‐1β, TNF‐α, IL‐8, IL‐6, and IFN‐β (Tsirigotis‐Maniecka et al. 2023; Kotb et al. 2024). The NF‐κB signaling pathway is a key regulator of biochemical and immunological responses in eukaryotic cells, responding to diverse stimuli such as pathogens, toxins, cytokines, oxidative stress, and environmental changes (Chawla et al. 2021). Activation of NF‐κB involves a multi‐step process including phosphorylation, ubiquitination, and degradation of inhibitory IκB subunits, leading to NF‐κB translocation from the cytoplasm to the nucleus (Li and Lin 2008). In its inactive state, NF‐κB is bound to IκB in the cytoplasm; upon IκB phosphorylation and degradation, NF‐κB is released and translocates to the nucleus where it binds to specific gene sequences, initiating transcription of pro‐inflammatory cytokines (Strickland and Ghosh 2006). NF‐κB regulates numerous genes involved in inflammation and is highly activated in diseased tissues characterized by inflammation, such as multiple sclerosis, inflammatory bowel disease, psoriasis, and asthma, underscoring its central role in inflammatory processes (Gilmore 2008; Abukhalil et al. 2025).

Polyphenols have been shown to activate NF‐E2‐related factor pathways, potentially modulating NF‐κB activity (Mueller et al. 2013), suggesting *A. eupatoria* may downregulate pro‐inflammatory cytokine mRNA expression like TNF‐α and IL‐1β in LPS‐exposed blood cells (Hougee et al. 2005). Flavonoids and glycosides from *A. eupatoria* , including luteolin and apigenin, suppress NF‐κB transcriptional activity in stimulated monocytes (Nicholas et al. 2007) and macrophages (Yang et al. 2021), indicating a mechanism for anti‐inflammatory effects. *Agrimonia* species exhibit anti‐inflammatory potential (Vogl et al. 2013), though exact mechanisms are not fully understood. Recent findings indicate *A. eupatoria* extracts reduce NF‐κB activity by inhibiting nuclear translocation (Huzio et al. 2022), pointing to a specific pathway through which this plant exerts its anti‐inflammatory actions.

## 5 Limitation and Further Study

This research is subject to certain limitations, notably time constraints, budgetary restrictions, and limited access to materials and instruments, which may affect the comprehensiveness and validity of the results. A major limitation of this study is the small sample size, with only 30 rats used in the experiments. This limited sample size increases the likelihood of false positives and false negatives, potentially leading to statistically insignificant results and reducing the study's power to detect meaningful differences. In future studies, the number of rats should be increased to obtain more accurate and significant results.

The study was limited by testing only two dose concentrations (250 mg/kg and 500 mg/kg). A wider range of concentrations would provide a more accurate determination of the effective dose range.

The study's reliance on absolute ethanol as the only necrotizing agent to induce gastric ulcers limits its scope. Other agents (e.g., indomethacin, aspirin, acetic acid, NSAIDs) and methods (e.g., pyloric ligation, stress‐induced lesions) could be explored in future research to provide a more comprehensive understanding of gastric ulcer induction and treatment.

## 6 Conclusion

In conclusion, *A. eupatoria* leaves showed substantial antiulcer effects reliant on dosage, contrary to alcohol—convinced stomach grazes animal ideal. Gastro‐protective effects of *A. eupatoria* could be linked to the active straight radical scavenging action, the augmentation of cellular antioxidant accomplishments such as SOD and CAT levels, besides the lessening of lipid peroxidation. Rats treated with *A. eupatoria* exhibited lower gastric lesions and ulcer index than the ulcer control group, as demonstrated by evaluations of PAS expression, gastric mucus content, and anti‐radical enzymes in gastric tissue.

## Author Contributions

**Suhayla Hamad Shareef:** conceptualization (lead), data curation (lead), formal analysis (lead), investigation (supporting), methodology (supporting), project administration (lead), resources (lead), software (lead), supervision (lead), validation (lead), visualization (lead), writing – original draft (lead), writing – review and editing (lead). **Nahla Kamal Asaad:** resources (equal), software (equal), visualization (equal), writing – review and editing (equal). **Noor Ali Gheni:** investigation (equal), visualization (supporting), writing – review and editing (supporting). **Derin Nabaz Fisal:** data curation (supporting), validation (equal), writing – review and editing (supporting). **Nabaz Fisal Shakir Agha:** project administration (equal), validation (equal), writing – review and editing (equal). **Ronak Tahr Ali:** data curation (equal), writing – review and editing (equal). **Mahmood Ameen Abdulla:** conceptualization (lead), data curation (lead), investigation (equal), methodology (equal), project administration (lead), resources (equal), supervision (lead), visualization (equal), writing – original draft (lead), writing – review and editing (equal).

## Consent

The authors have nothing to report.

## Conflicts of Interest

The authors declare no conflicts of interest.

## Data Availability

Data supporting the current study is available on request.
